# *Trichuris suis* and *Oesophagostomum dentatum* Show Different Sensitivity and Accumulation of Fenbendazole, Albendazole and Levamisole *In Vitro*

**DOI:** 10.1371/journal.pntd.0002752

**Published:** 2014-04-03

**Authors:** Tina V. A. Hansen, Peter Nejsum, Christian Friis, Annette Olsen, Stig Milan Thamsborg

**Affiliations:** Department of Veterinary Disease Biology, Faculty of Health and Medical Sciences, University of Copenhagen, Frederiksberg C, Denmark; Swiss Tropical and Public Health Institute, Switzerland

## Abstract

**Background:**

The single-dose benzimidazoles used against *Trichuris trichiura* infections in humans are not satisfactory. Likewise, the benzimidazole, fenbendazole, has varied efficacy against *Trichuris suis* whereas *Oesophagostomum dentatum* is highly sensitive to the drug. The reasons for low treatment efficacy of *Trichuris* spp. infections are not known.

**Methodology:**

We studied the effect of fenbendazole, albendazole and levamisole on the motility of *T. suis* and *O. dentatum* and measured concentrations of the parent drug compounds and metabolites of the benzimidazoles within worms *in vitro*. The motility and concentrations of drug compounds within worms were compared between species and the maximum specific binding capacity (B_max_) of *T. suis* and *O. dentatum* towards the benzimidazoles was estimated. Comparisons of drug uptake in living and killed worms were made for both species.

**Principal findings:**

The motility of *T. suis* was generally less decreased than the motility of *O. dentatum* when incubated in benzimidazoles, but was more decreased when incubated in levamisole. The B_max_ were significantly lower for *T. suis* (106.6, and 612.7 pmol/mg dry worm tissue) than *O. dentatum* (395.2, 958.1 pmol/mg dry worm tissue) when incubated for 72 hours in fenbendazole and albendazole respectively. The total drug concentrations (pmol/mg dry worm tissue) were significantly lower within *T. suis* than *O. dentatum* whether killed or alive when incubated in all tested drugs (except in living worms exposed to fenbendazole). Relatively high proportions of the anthelmintic inactive metabolite fenbendazole sulphone was measured within *T. suis* (6–17.2%) as compared to *O. dentatum* (0.8–0.9%).

**Conclusion/Significance:**

The general lower sensitivity of *T. suis* towards BZs *in vitro* seems to be related to a lower drug uptake. Furthermore, the relatively high occurrence of fenbendazole sulphone suggests a higher detoxifying capacity of *T. suis* as compared to *O. dentatum*.

## Author Summary

The human whipworm *Trichuris trichiura* is together with the roundworm *Ascaris lumbricoides* and the hookworms *Ancylostoma duodenale* and *Necator Americanus* the most common intestinal worms worldwide. Together they place more than 5 billion people at risk of infection. The current global control strategy against these worms is regular administration of anthelmintic drugs, mostly albendazole and mebendazole, both belonging to the drug-class benzimidazoles. Both drugs have a low effect against *T. trichiura* infections, but the reasons for this are not known. We evaluated the *in vitro* effect of two benzimidazoles; i.e., albendazole, fenbendazole, and another type of anthelmintic, levamisole, on the whipworm (*T. suis*) and the nodular worm (*Oesophagostomum dentatum*) of the pig. *Oesophagostomum dentatum* is highly sensitive towards benzimidazoles in comparison to *T. suis*. We measured and compared the drug uptake in both species in both living and killed worms. Our results suggest that the reason for the difference in sensitivity is due to a lower drug uptake into *T. suis* as compared to *O. dentatum*. Furthermore, *T. suis* was able to metabolise fenbendazole into an inactive metabolite to a much larger extent than *O. dentatum*, suggesting a higher detoxifying capacity of *T. suis* as compared to *O. dentatum*.

## Introduction

The whipworm *Trichuris trichiura* has been estimated to infect 600 million people worldwide resulting in an estimated 1.6–6.4 million disability adjusted life-years lost globally [1]. The current control strategy against *T. trichiura* and other soil-transmitted helminths (STHs) is administration of single-dose anthelmintic drugs [1], [2]. The benzimidazoles (BZs) i.e. albendazole (ALB) and mebendazole (MBD) are widely used in large-scale control programs where they are administered regularly, at a dosage of 400 mg (ALB) or 500 mg (MBD) [2]. However, the efficacy of single-dose BZ against *T. trichiura* is not satisfactory. A meta-analysis of 20 randomized, placebo-controlled trials reported an average cure rate (CR) of 28% for ALB (400 mg) and 36% for MBD (500 mg) [3]. Other randomized controlled trials have reported similar low CR and egg reduction rates (ERR) ranging from 31.5–40.3% (CR) and 9.8–54.0% (ERR) for ALB and 22.9–66.7% (CR) and 18.8–81.0% (ERR) for MBD [4]–[7].

The use of the *T. muris*-mouse model for estimating drug efficacy on *T. trichiura* is well established [8]–[11]. *Trichuris suis* is regarded a different but closely related species to *T. trichiura* [12], [13], hence, *T. suis* can be considered a valid model for *T. trichiura*. Another BZ, fenbendazole (FBZ) has shown poor efficacy against *T. suis* infection in pigs when administered as a single-dose [14], therefore the *T. suis*-pig model and FBZ may be considered an interesting alternative for studying low treatment efficacy of *Trichuris* spp. In one controlled trial an oral dose as high as 15 mg/kg, three times the recommended dose of 5 mg/kg for other pig nematodes, was required to obtain a worm count reduction (WCR) of 96.7% [14]. In another controlled study the same oral dose resulted in only a 65.1% reduction in worm burden and a dose of 30 mg/kg resulted in an efficacy of 96.6% [15]. Multiple doses of FBZ (3 mg/kg per day for 3 consecutive days) have shown varied efficacy against *T. suis* in controlled tests ranging from 66% [16] to 99.8% [14], [17] in WCR. The current recommendation for treatment of *T. suis* infections in pigs with FBZ is either a single dose of 25 mg/kg, or a long-term treatment where the recommended therapeutic dose is distributed over 7 days [18], [19].

Another nematode of the pig is the nodular worm, *Oesophagostomum dentatum* which in the adult stage, opposed to *T. suis*, is highly sensitive to FBZ. An oral dose level as low as 0.25 mg/kg has shown an efficacy of 99.9% and doses of 1, 2.5 and 3.5 mg/kg FBZ have resulted in efficacies of 100% in controlled tests based on worm counts [20], [21]. *Trichuris suis* and *O. dentatum* both inhabit the lower part of the intestine namely the caecum and the colon [22]–[25], but in their adult stage, their microhabitat varies significantly. The thin anterior part of *T. suis* is embedded in the mucosa creating a tunnel-like construction of epithelial cells whereas the thicker posterior part of the body is protruding freely into the lumen [26]. In contrast to *T. suis*, the adult stage of *O. dentatum* is not attached to the mucosa but roams freely in the intestinal lumen [27], [28].

Levamisole (LEV), belonging to another class of anthelmintics, the imidazothiazoles, was introduced in 1968 [29] and has like BZs been used against parasitic infections in both animals and humans. In order for BZs and imidothiazoles to exert their pharmacological effect, they need to reach their specific receptors within the target parasites i.e. BZs bind to beta-tubulin [30] and the imidazothiazoles to acetylcholine-gated channels [29], [31]. Passive diffusion through the external surface has been proposed as the main pathway of BZs (i.e. FBZ, oxfendazole (OXF) and triclabendazole sulphoxide (TCBZSO)) in the three main classes of helminth parasites represented by: *Moniezia benedeni* (cestode), *Fasciola hepatica* (trematode) and *Ascaris suum* (nematode) [32]. The uptake of LEV has likewise been demonstrated to occur via a transcuticular mechanism in *A. suum*, but was observed to take place in four distinct stages, thus suggesting a non-passive up-take mechanism [33]. Once inside an organism, drugs are generally being metabolised. However, our knowledge of the metabolism of anthelmintics in helminths is very limited, although drug metabolising enzymes are well described in mammals and serve as an efficient defense mechanism against potential harmful substances. In brief drugs are (if not excreted unchanged) biotransformed by unique enzymes into more polar compounds that are easier to excrete by the organism in metabolic reactions named phase I-III. In mammals the major phase I reaction is oxidation catalysed by cytochrome P450 superfamily (CYPs) [34]. For many years attempts to detect CYPs in parasitic nematodes were unsuccessful [35] but with the discovery of 75 predicted CYP genes in the free-living nematode *Caenorhabditis elegans* as well as genomic and transcriptomic-based predictions of proteins produced by helminths, the knowledge has improved [36]. The ability of parasitic helminths to metabolise anthelmintics may serve as an advantageous defence mechanism. Previously, the first step of phase I oxidation of ALB into albendazole sulphoxide (ALBSO) (sulphoxidation) has been reported for *F. hepatica*, *M. expansa*, *A. suum* [37], *Dicrocoelium dendriticum* [38] and *Haemonchus contortus* [39]. This metabolite has a lower pharmacological activity than the parent compound [40] and lower effect on nematode motility [41]. The second step of ALB oxidation (sulphonation) into albendazole sulphone (ALBSO_2_) was reported for *D. dendriticum* [38]. A similar sulphonation process has been reported for *F. hepatica* exposed to triclabendazole sulphoxide (TCBZSO) *in vitro* [42]. To the best of our knowledge no studies has been conducted on the metabolism of FBZ within parasitic nematodes. Comparative *in vitro* studies of the oxidative metabolism of FBZ by hepatic microsomal fractions from a variety of vertebrate species showed that all species readily produced the sulphoxide metabolite ( = oxfendazole, OXF) and the sulphone metabolite fenbendazole sulphone (FBZSO_2_) [43]. Oxfendazole is a widely used anthelmintic whereas FBZSO_2_, similar to ALBSO_2_, are considered pharmacological inactive [40], [44].

We find the different sensitivity of *T. suis* and *O. dentatum* to FBZ *in vivo* highly interesting because these two species are located in the same compartment of the intestine and thus theoretically exposed to similar concentrations of drugs. We speculate that the difference in sensitivity may be related to differences in uptake and/or metabolism of the drug inside the worms. We hypothesized that the reason for a low or variable treatment efficacy of *T. suis* infections may be due to a lower drug uptake and/or a higher drug metabolism of *T. suis* in comparison to *O. dentatum*. The aim of this study was therefore to examine the motility of *T. suis* and *O. dentatum* adult worms *in vitro* when exposed to FBZ, ALB and LEV and to assess whether these drugs accumulate in the same concentrations within the two species.

## Materials and Methods

### 2.1 Drugs

Fenbendazole, ALB and LEV were purchased from Sigma-Aldrich (Schnelldorf, Germany), and stock solutions of the drugs (100.000 µM) were prepared in 100% dimethylsulfoxid (DMSO) (Sigma-Aldrich, Schnelldorf, Germany) and stored at 5°C until use within 1 week.

### 2.2 Experimental animals and parasite infections

Fourteen pigs were purchased and acclimatized for 1 week prior to experimental infection. The animals had free access to water and were fed restrictively, according to national feeding requirements. For the FBZ *in vitro* assay, six pigs were orally infected by stomach tube with 2,000 embryonated *T. suis* eggs (kindly provided by *Parasite Technologies A/S*, Hørsholm, DK) and two pigs with 5,000 L3 *O. dentatum* larvae (CEP-strain). The CEP-strain was originally isolated from a farm with no prior use of anthelmintics according to the owner [45], and was later characterized as FBZ susceptible [21]. The *T. suis* isolate has been used in an *in vivo* study where experimentally infected pigs were exposed to repeated administration of FBZ (i.e. 5 mg/kg given orally on three consecutive days). Worm count reductions of 51.5 and 98.5% were obtained 24 hours after single and triple dose treatments, respectively; therefore, this isolate was considered FBZ susceptible. For the ALB and LEV *in vitro* assay, 3 pigs were infected with 5,000 embryonated *T. suis* eggs and 3 pigs with 4,000 L3 *O. dentatum* larvae (same strains as above). Due to practicalities the experimental infections for ALB and LEV were performed after the FBZ assay. Patency of infections was confirmed by faecal egg count (EPG) using the modified McMaster technique [46].

### 2.3 Ethic statement

The current study was approved by the Experimental Animal Unit, University of Copenhagen, (Denmark) based on national regulations from the Danish Animal Experiments Inspectorate (permission no. 2010/561-1914, C5).

### 2.4 Recovery of nematodes

For the FBZ *in vitro* assay, the *O. dentatum* infected pigs were euthanized at day 40 post infection (p.i.) and the *T. suis* infected pigs at day 63 p.i. For the ALB and LEV *in vitro* assay the *O. dentatum* and the *T. suis* infected pigs were euthanized at day 28 and 49 days p.i., respectively. Adult *O. dentatum* were isolated from the intestinal content according to Slotved et al. [47] and adult *T. suis* were collected from the intestine by manual plucking. Both parasite species were washed following a common washing procedure which consisted of 4 consecutive washing steps (each 15 min. in 39°C Hanks Balanced Salt Solution (HBSS)) followed by 4 consecutive washing steps (each 60 min. in 39°C RPMI-1640 medium). Both the HBSS and RPMI-1640 media were supplemented with 1% (v/v) amphotericin B-penicillin-streptomycin solution (10,000 U/ml penicillin, 10,000 µg/ml streptomycin, 25 µg/ml amphotericin B) and 0.5% (v/v) gentamicin (10 mg/ml) (All media, antibiotics and anti-mycotic were purchased from Life Technologies, Naerum, DK).

### 2.5 *In vitro* motility assay

Since FBZ concentrations above 30 µM precipitated during incubation, we tested the following concentrations of FBZ and ALB: 0.01, 0.1, 1, 10 and 30 µM.

Final concentrations of LEV included 0.01, 0.1, 1, 10 and 200 µM. All dilutions contained DMSO (2% v/v) and were made in RPMI-1640 medium supplemented with antibiotics and fungicide as described for the washing procedure. Thirty worms of each species selected at random were placed in a large petri dish (Th. Geyer, Roskilde, DK) containing 40 ml of each of the dilutions described above. Each concentration was tested in triplicate, thus for each drug and each concentration a total of 90 worms were used. Worms incubated in RPMI-1640 with DMSO 2% (v/v) without anthelmintics served as controls. All worms were incubated at 39°C (5% CO_2_, 21% O_2_, 90% relative humidity) for 24 or 72 hours. In the motility assay, 21 worms (i.e. 7 worms from each petri dish) of both species were scored by stereomicroscope at 6.3× magnification according to motility grades specific for each species. The motility of *T. suis* was graded as follows: 3: normal motility (movement of the whole body), 2: low motility (slower movement of the whole body), 1: very low motility (movement of the anterior part only), 0: no movements. The motility of *O. dentatum* was graded as follows: 3: normal motility (swimming), 2: low motility (slow swimming or jerking movements), 1: very low motility (only movement of the anterior tip of the body), 0: no movements. All motility measurements were blinded except for worms incubated in FBZ, due to lack of resources.

### 2.6 Comparison of *in vitro* drug uptake in living and killed nematodes

In order to compare the accumulation of drugs in living and killed worms, a number of worms obtained after the common washing procedure was killed by freezing (liquid nitrogen for 1 min.) and thawed at 5°C. Thirty living and 30 killed worms of each species were then incubated for 24 hours in FBZ, ALB or LEV at a final concentration of 10 µM in RPMI-1640 medium with DMSO (2% v/v) using the same conditions as described above. All incubations were performed in triplicates.

### 2.7 Preparation of nematodes and HPLC analysis

After motility measurements and the 24 hour incubation period of living and killed *T. suis* and *O. dentatum*, all worms were carefully rinsed in 50 ml HBSS for a maximum of 30 sec. The *in vitro* assay with FBZ was conducted first, and since the drug concentration within worms was unknown, all worms from each incubation concentration were pooled into one sample to ensure a detectable drug level. Subsequently, triplicates were made for worms incubated in each of the five concentrations of ALB and LEV. After rinsing, worms were transferred to pre-weighed Eppendorf vials, frozen in liquid nitrogen and kept at −20°C until HPLC-analysis.

Vials with worms were thawed and dried under phosphorous pentoxide until constant weight. Each vial with dried worm (10–50 mg) was mixed with 200 µl 0,05M phosphate buffer (pH 7.4) with internal standard (see below). After gentle homogenization with a plastic pestle another 200 µl buffer was added and the homogenization repeated before addition of 400 µl 6M guanidine HCl. The sample was vortexed for 1 minute and left at 20°C for 15 minutes before centrifugation at 8000× g for 10 minutes. The supernatant was transferred to a clean tube and an additional 400 µl of 6M guanidine HCl was added to the sample residue. The procedure was repeated and the two supernatants were pooled and loaded on an activated cartridge (Oasis HLB, 60 mg, 3 mL). The cartridge was activated with 2 mL methanol (100%) followed by 2 mL of water. The loaded cartridge was washed with 2 mL 5% methanol and dried under vacuum for 1 minute, before eluting the analyte with 2 mL methanol. The eluate was evaporated under air at 37°C and the residuum was dissolved in 100 µL 50% methanol and centrifuged at 8000× g before 50 µL were injected into the HPLC-system. Standards in phosphate buffer and guanidine HCl were run in parallel. Concentration of analyte in worms was expressed as µg per g dry worm.

The HPLC system was equipped with an autosampler, 2 HPLC pumps, and a UV detector. HPLC conditions for FBZ, ABZ and LEV are described below:

#### Fenbendazole

No internal standard was used in the FBZ analysis. The UV detector was set to 294 nm. Separation of analytes was accomplished at 30°C on a Novapak C18 (5 µ, 15 cm). The mobile phase consisted of a gradient mixed from acetonitrile and 0.025*M* ammonium acetate (pH 7.2) at a flow rate of 1 ml/min. The proportion of acetonitrile was 30% acetonitrile for the first 3 minutes, progressing linearly to 40% at 3.5 minutes, held constant at 40% until 11 minutes and finally reduced to 30% at 11.5 min for the remaining run time of 17 minutes. Retention times for FBZ, OXF and FBZSO_2_ were 13 min, 2.5 min and 4.5 min, respectively. Standards of FBZ, OXF and FBZSO_2_ were prepared from stock solutions in DMSO. Peak area of each analyte was used to calculate concentration. The limit of quantification for FBZ, OXF and FBZSO_2_ was 2 ng/mg dry worm.

#### Albendazole

FBZSO_2_ was used as internal standard in a concentration of 1 µg/ml. The UV detector was set to 290 nm. Separation of analytes was accomplished at 30°C on a Novapak C18 (5 µ, 15 cm).The mobile phase consisted of a gradient mixed from acetonitrile and 0.025*M* ammonium acetate (pH 7.2) at a flow rate of 1 ml/min. The proportion of acetonitrile was 25% acetonitrile for the first 2 minutes, progressing linearly to 50% at 2.5 minutes, held constant at 50% until 9 minutes and finally reduced to 25% at 9.5 min for the remaining run time of 17 minutes. Retention times for ALB, ALBSO, ALBSO_2_ and FBZSO_2_ (IS) were 9 min, 2 min, 3.5 min and 7 min, respectively. Standards of ALB, ALBSO, ALBSO_2_ were prepared from stock solutions in DMSO. Peak high of analyte to internal standard was used to calculate the concentration of analyte. The limit of quantification for ALB, ALBSO and ALBSO_2_ was 2, 0.1, 5 ng/g dry worm, respectively.

#### Levamisole:

Lidocaine was used as internal standard in a concentration of 5 µg/ml. The UV detector was set to 214 nm. Separation of analyte was accomplished at 30 C on a X-bridge C18 (5 µ, 15 cm). The mobile phase consisted of 25% acetonitrile and 75% phosphoric acid (0.1%) containing 0.1% octansulphone acid at a flow rate of 1 ml/min. Retention times for levamisole and lidocaine (IS) were 6.5 min and 10 min, respectively. Standards of LEV and lidocaine (IS) were prepared from stock solutions in water. Peak high of analyte to internal standard was used to calculate the concentration of analyte. The limit of quantification for LEV was 2 ng/g dry worm.

### 2.8 Statistical analysis

All motility scores were normalized into percentages relative to controls within species. For each drug the effect of all factors (species, time and log_concentration) and biological meaningful interactions between the factors were tested for statistical significance (*P*<0.05) using Analysis of Covariance (ANCOVA) with variance heterogeneity using SAS version 9.3 and JMP version 8 (SAS Institute, Cary, North Carolina). Due to significant effects of time, the effect of drug concentrations in the media on the relative motility of the two species was then calculated for 24 and 72 hours separately. Variance heterogeneity was used since the variances between the species were different. Total drug concentrations (parent compound and its metabolites) in living and killed worms of each species were compared using Student's t-test with variance heterogeneity (JMP version 8). Drug concentrations in worms exposed to 5 concentrations of FBZ and ALB were compared using the model ‘One site fit total and nonspecific binding’ (GraphPad Prism 5, GraphPad Software, San Diego, California) which calculates the parameter estimates K_d_ and B_max_ by the following equation: Y = B_max_*X/(K_d_+X)+NS*X+background. X and Y are drug concentrations in media and worms, respectively. K_d_ is the concentration of a ligand which is needed in order to achieve half-maximum binding at equilibrium. B_max_ is the maximum specific binding, thus giving the maximum binding capacity of an object or organism. NS is the slope of non-specific binding. Background and NS was constrained to 0 since no binding was observed when measuring the negative controls. The difference of K_d_ and B_max_ between the species was evaluated on a significance level of α = 0.05. Drug concentrations in worms exposed to LEV were compared using Student's t-test (JMP version 8) because only the two highest concentrations yielded detectable levels within the worms. Thus, concentration difference between and within species was evaluated when worms were exposed to 10 and 200 µM LEV respectively. For each drug, all data sets were tested for normality.

## Results

### 3.1 Motility

**Figure 1:**
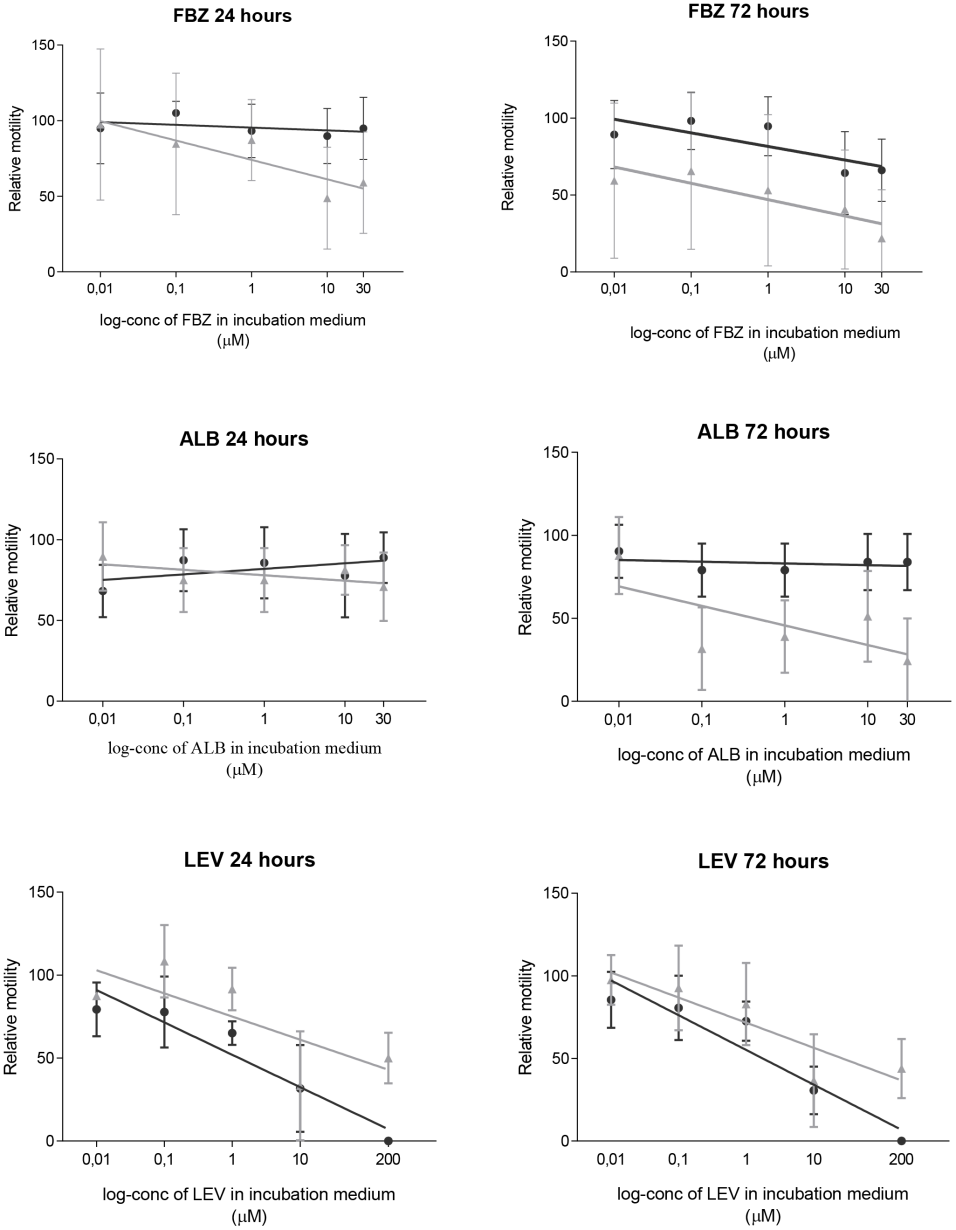
Mean relative motility (± SD, *n* = 21) and a tendency line for *Trichuris suis* (dark gray circle) and *Oesophagostomum dentatum* (light gray triangle) after exposure to FBZ, ALB, and LEV for 24 and 72 hours.

The relative motility of *T. suis* and *O. dentatum* after exposure to FBZ, ALB and LEV for 24 and 72 hours are presented in Fig. 1. No significant difference in motility between species was observed with increasing concentration over time for FBZ, ALB or LEV (species*time*log_concentration). The motility of *T. suis* was found to be less affected by time (24 vs. 72 h) than *O. dentatum* when exposed to FBZ (*P* = 0.015) and ALB (*P*<0.0001), but not LEV (species*time). The motility of *T. suis* was significantly less affected than that of *O. dentatum* after 24 hours incubation in FBZ (*P* = 0.003) but not 72 hours (*P* = 0.73) (species*log_concentration). Although the interaction was not significant after 72 hours, the motility of *T. suis* was still significantly less affected than the motility of *O. dentatum* (*P*<0.0001) (species) and the increasing concentration of FBZ resulted in a significant motility decrease for both species (*P* = 0.012) (log_concentration). When exposed to increasing concentrations of ALB, the motility of *T. suis* was less affected than *O. dentatum* after both 24 hours (*P* = 0.003) and 72 hours (*P*<0.0001) (species*log_conc). The opposite was observed for increasing concentrations of LEV where the motility of *T. suis* was reduced more than *O. dentatum* after 24 (*P*<0.007) and 72 hours (*P*<0.007) (species*log_conc).

### 3.2 Drugs concentrations within living and killed worms

**Figure 2:**
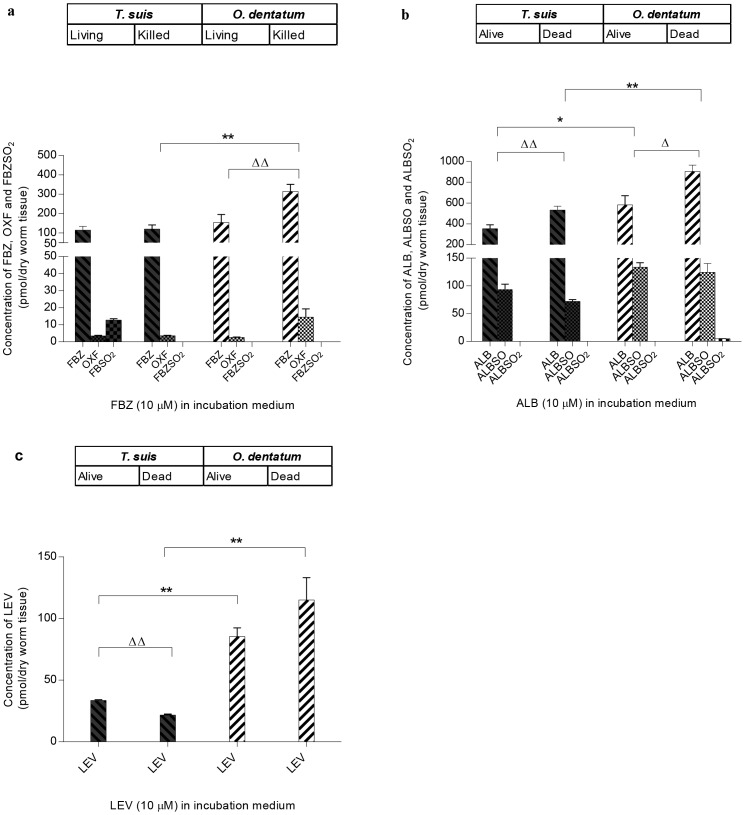
Mean concentration (± SD, *n* = 3, each replicate consist of 30 worms) of a) FBZ, OXF and FBZSO_2_, b) ALB, ALBSO and ALBSO_2_ and c) LEV measured in living and killed *Trichuris suis* and *Oesophagostomum dentatum* after incubation for 24 hours in 10 µM of each of the parent compound. Significant difference in total concentration (parent compound+metabolites) between species is indicated with *, significant difference within the species (living and killed) is indicated with ▵. *P*-values were obtained using Student's t-test with variance heterogeneity. *P<0.05 and **P<0.01 and ^▵^ *P*<0.05 and ^▵▵^ *P*<0.01.

The mean concentrations of the parent compounds FBZ, ALB and LEV and the metabolites of FBZ (OXF, FBZSO_2_) and ALB (ALBSO, ALBSO_2_) in living and killed worms after incubation in 10 µM of the drug for 24 hours are shown in Fig. 2. In general, the total drug concentrations within both living and killed worm species varied according to type of drug (Fig. 2a, 2b, 2c), with ALB and its metabolite ALBSO occurring at the highest concentration level followed by FBZ and its metabolites and LEV. When incubated in ALB and LEV, the total drug concentrations were found to be significantly lower in *T. suis* than *O. dentatum* and this was observed for both living (ALB: *P* = 0.02, LEV: *P* = 0.02) and killed (ALB: *P* = 0.002, LEV: *P* = 0.008) worms. In both living and dead worms, the total concentration of FBZ and its metabolites was found to be lower in *T. suis* than *O. dentatum*. For the dead worms, the difference was significant (*P* = 0.004) but did not reach significance for living worms (131.1±17.1 pmol/mg dry worm tissue vs. 155.8±33.3 pmol/mg dry worm tissue for *T. suis* and *O. dentatum*, respectively).

For *O. dentatum* the concentration of drug was higher in killed worms as compared to living worms for all three anthelmintics, and the difference was found to be significant when incubated in FBZ (*P* = 0.006) and ALB (*P* = 0.011). For *T. suis* no difference between the living and the killed was observed when incubated in FBZ, whereas the anthelmintic concentration was significantly higher within killed worms when incubated in ALB (*P* = 0.009) and significantly lower when incubated in LEV (*P*<0.001). The mean concentrations of OXF in living and killed worms, respectively, were found to be 3.4 and 3.5 pmol/mg dry worm tissue for *T. suis* and 2.6 and 14.4 pmol/mg dry worm tissue for *O. dentatum*. The pharmacological inactive metabolite FBZSO_2_ (mean: 12.7 pmol/mg dry worm tissue) was only observed in living *T. suis* and amounted 9.7% of the total anthelmintic concentration measured within the worms. The mean concentrations of ALBSO in living and killed worms were 93.8 and 71.9 pmol/mg dry worm tissue, respectively, for *T. suis* and 133.8 and 124.4 pmol/mg dry worm tissue for *O. dentatum*. Only trace amount of ALBSO_2_ (4.71 pmol/mg dry worm tissue) were measured in killed *O. dentatum*.

### 3.3 Concentrations of total drug within living worms exposed to different drug levels

**Figure 3:**
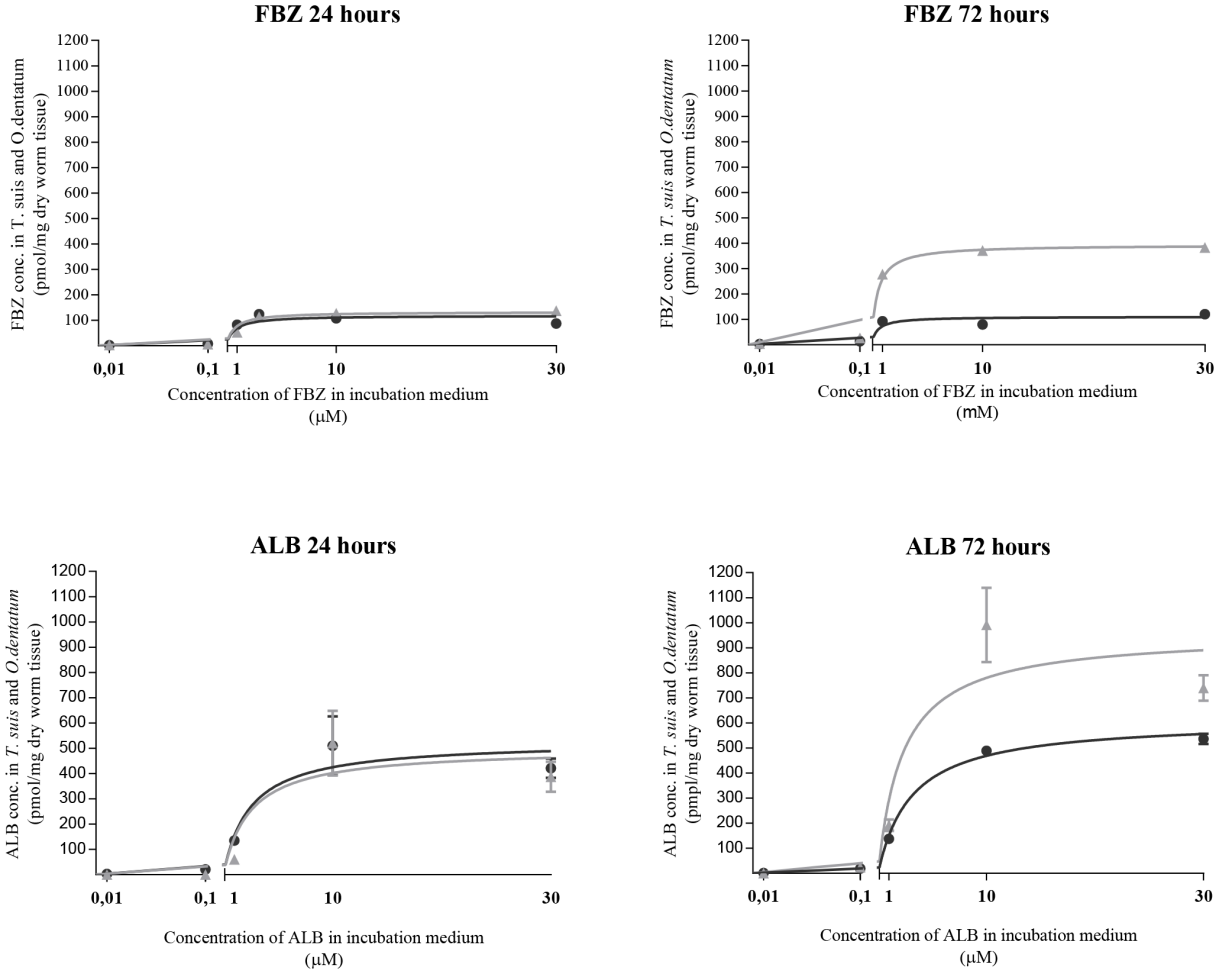
Non- specific binding of FBZ and ALB accumulated inside living *Trichuris suis* (dark gray circle) and *Oesophagostomum dentatum* (light gray triangle) after incubation in 0.01, 0.1, 1,10 or 30 µM FBZ or ALB for 24 and 72 hours. Mean concentrations (pmol/mg dry worm tissue) of ALB (± SD, *n* = 3) are shown, while the values of FBZ represents one sample only for each concentration at 24 and 72 hours, respectively.

**Figure 4:**
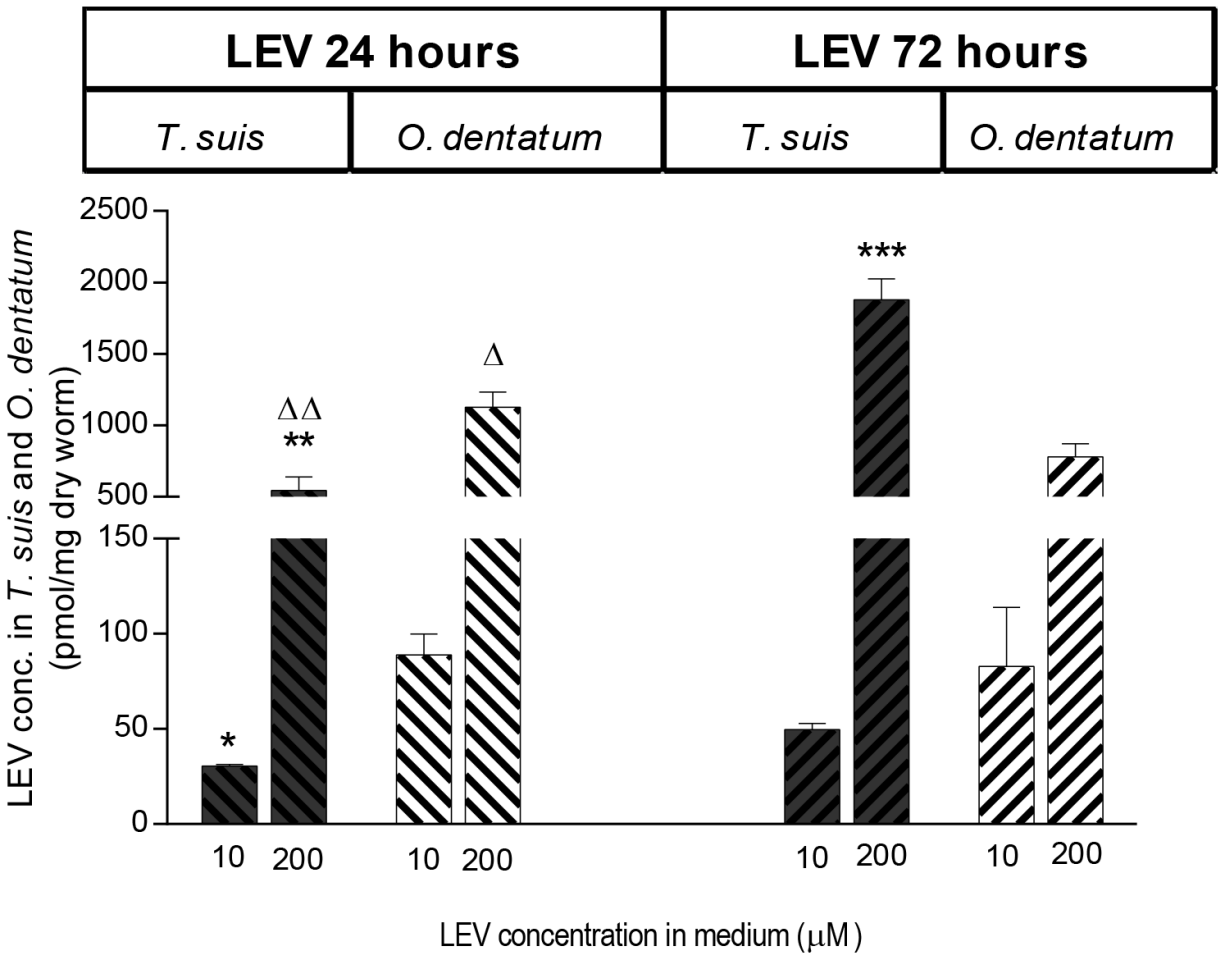
Mean concentration of LEV (± SD, *n* = 3) measured in living *Trichuris suis* and *Oesophagostomum dentatum* after incubation in 10 or 200 µM LEV for 24 and 72 hours. Statistically different concentration between species when exposed to either 10 or 200 µM LEV for 24 and 72 hours, respectively are indicated with: *P<0.05, **P<0.01 and ***P<0.001. Statistically different concentration values within the species between 24 and 72 hours are indicated with: ^▵^ *P*<0.05 and ^▵▵^ *P*<0.01.

The concentration of FBZ and ALB inside living *T. suis* and *O. dentatum* after incubation in 0.01, 0.1, 1, 10 and 30 µM of FBZ and ALB for 24 and 72 hours is shown in Fig. 3. The K_d_ and B_max_ values for each species at 24 and 72 hours are given in Table 1. For both anthelmintic drugs no significant difference in the K_d_ – values were observed between the species neither after 24 or 72 hours of incubation. The B_max_ – values were similar for the two species after 24 hours exposure to both BZs, but after 72 hours incubation, these were significantly lower for *T. suis* than *O. dentatum* when exposed to FBZ (*P*<0.0001) and ALB (*P* = 0.033). The concentrations of LEV found within the worms after exposure to 0.01, 0.1, 1, 10 and 200 µM LEV for 24 and 72 hours were only above the detection limit when exposed to the two highest concentrations (Fig. 4). The concentrations of LEV found within the worms were significantly lower in *T. suis* than *O. dentatum* when incubated in 10 and 200 µM for 24 hours (*P* = 0.01, *P* = 0.0009). When incubated in 200 µM for 72 hours the concentration of LEV was higher in *T. suis* (452.5 ng/mg dried worm tissue) than in *O. dentatum* (187.9 ng/mg dried worm tissue) (*P*<0.0001). The concentration of LEV within *T. suis* thus increased significantly with incubation time (P<0.0001) when incubated in 200 µM LEV, whereas the concentration was lower after 72 hours than 24 hours incubation within *O. dentatum* (*P* = 0.02).

**Table 1:** Comparison of the binding constant at equilibrium (K_d_) and the maximum specific binding capacity (B_max_) of *Trichuris suis* and *Oesophagostomum dentatum* incubated in FBZ and ALB for 24 and 72 hours.

	*T. suis*		*O. O. dentatum*	
	24 hours	72 hours	24 hours	72 hours
FBZ				
K_d_	0.37	0.33	1.36	0.54
B_max_	110.9	106.6^a^	147.0	395.2^a^ ***
ALB				
K_d_	2.03	3.57	2.87	2.28
B_max_	514.1	612.7^b^	513.9	958.1^b^ *

K_d_ is given in µM and B_max_ in pmol/mg dry worm tissue. Comparisons are made between nematode species for K_d_ – and B_max_ -values for each time point and *P*-values indicated for values with same superscript:

* *P*<0.05,

*** *P*<0.0001,

a, b : comparison of B_max_-values after exposure to FBZ and ALB respectively, for 72 hours.

### 3.4 Concentrations of drug metabolites in worms exposed to different levels of anthelmintics

**Figure 5:**
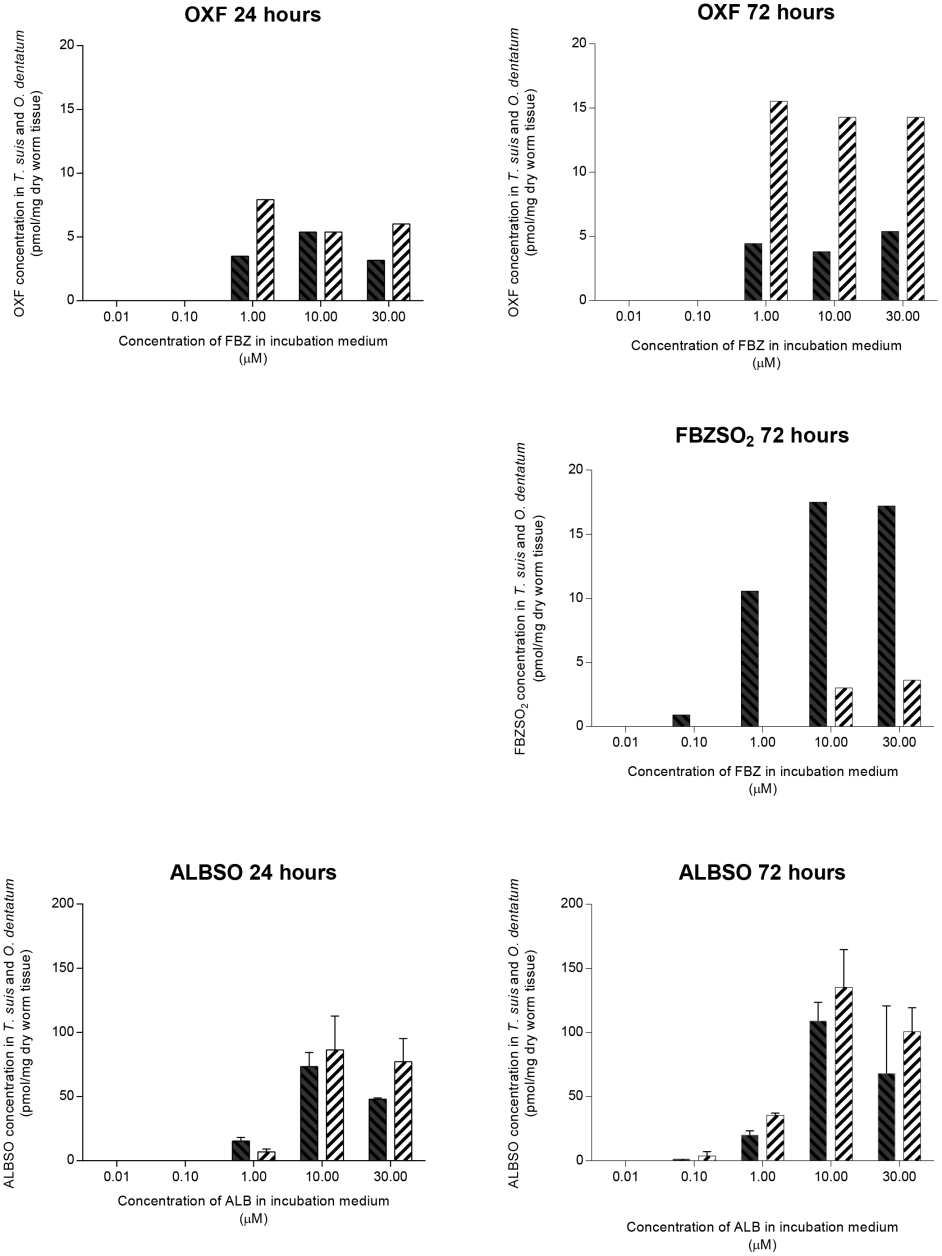
Concentrations of OXF, FBZSO_2_ and mean concentrations of ALBSO (± SD, *n* = 3) measured in living *Trichuris suis* (black columns) and *Oesophagostomum dentatum* (hatched columns) after incubation in 0.01, 0.1, 1, 10 or 30 µM FBZ (upper three graphs) or ALB (lower two graphs) for 24 and 72 hours.

The concentrations of the metabolites OXF, FBZSO_2_ and ALBSO measured within living *T. suis* and *O. dentatum* are given in Fig. 5. The concentrations of OXF and FBZSO_2_ within the two worm species were much lower than ALBSO (Fig. 5). Incubation concentrations below 0.1 µM of FBZ and ALB did not result in detectable levels of metabolites. The concentration of OXF within *T. suis* did not show a concentration or time dependent increase (3.2–5.4 pmol/mg dry worm tissue and 3.8–5.4 pmol/mg dry worm tissue after incubation periods of 24 and 72 hours, respectively) whereas a clear time dependent increase was observed for *O. dentatum* (5.4–7.9 pmol/mg dry worm tissue and 14.2–15.6 pmol/mg dry worm tissue after 24 and 72 hours, respectively). After 24 hours incubation the inactive metabolite FBZSO_2_ was only detected in *T. suis*. Results were inconsistent and are thus not given. After 72 hours incubation, FBZSO_2_ was detected within *T. suis* at an incubation concentration as low as 0.1 µM FBZ whereas FBZSO_2_ only appeared in *O. dentatum* when incubated in 10 and 30 µM. After 72 hours a concentration dependent formation of FBZSO_2_ (0.9–17.5 pmol/mg dry worm tissue) was measured within *T. suis* where it represented between 6–17.2% of the total drug concentration whereas in *O. dentatum* it only constituted 0.8–0.9%. For both species, the formation of FBZSO_2_ appeared to be both time- and concentration-dependent as consistent results only were obtained after 72 hours incubation. The ALBSO metabolite showed a clear tendency to reach a higher concentration within *O. dentatum* than *T. suis* when incubated for both 24 and 72 hours. The formation of ALBSO within the worms appeared to be both time- and concentration-dependent at incubation concentrations ranging from 0.1 µM to 30 µM. Incubation in 30 µM ALB resulted in ALBSO concentrations equal to or below the concentrations formed when incubated in 10 µM. The metabolite ALBSO_2_ was not detected within any of the two species. The metabolites OXF and ALBSO showed a clear tendency to reach a higher concentration level within *O. dentatum* than *T. suis* when incubated for both 24 and 72 hours, but in relation to the total drug concentration, the average proportion of the metabolites were approximately the same (OXF: *T. suis*; 4% at 24 hours and 3.6% at 72 hours; *O. dentatum*: 5.6% and 4%, ALBSO: *T. suis*; 11.1% and 13.8%, *O. dentatum*; 15% and 12.2%).

## Discussion

In the present work, we have combined worm motility with concentration measurements of drug-uptake and drug metabolism in two nematode species that inhabit the same part of the large intestine, but differ significantly in their intestinal microhabitat. Our results show that the motility of *T. suis* was less affected than the motility of *O. dentatum* when exposed to FBZ for 24 hours and ALB for 72 hours, thus indicating a lower sensitivity of *T. suis* as compared to *O. dentatum* towards these compounds. The maximum binding capacity of FBZ and ALB was significantly lower for *T. suis* than *O. dentatum* after 72 hours incubation and the total drug concentrations were significantly lower in living and killed *T. suis* as compared to *O. dentatum* when incubated in ALB. When living and killed worms were incubated in FBZ, only killed *T. suis* contained a significantly lower drug concentration than *O. dentatum*. However, collectively these results suggest *T. suis* to have a lower uptake of FBZ and ALB than *O. dentatum*. Furthermore, a relatively higher concentration of FBZSO_2_ was measured in *T. suis* than *O. dentatum*, thus suggesting a higher metabolism of FBZ (or OXF) into FBZSO_2_ in *T. suis*. Fenbendazole sulphone is considered anthelmintic inactive due to weak ovicidal activity and lack of inhibition of mammalian tubulin polymerization [44]. The equivalent sulphone metabolite of ALB, ALBSO_2,_ has not only shown complete loss of activity in both egg hatch inhibition assays and inhibition of mammalian tubulin polymerization but also decreased binding affinity to nematode tubulin [40]. Whether the latter also applies for FBZSO_2_ is not known but due to lack of polymerization inhibition, low ovicidal activity and assumed decreased binding affinity to nematode tubulin, FBZSO_2_ will in the following be considered “inactive”. However, caution must be taken. Due to uncertainty of detection levels within worms in the first trial, triplicates were not made for *T. suis* and *O. dentatum* incubated at different drug levels of FBZ (i.e. 0.01–30 µM). Although triplicates were not obtained, concentration agreement was found within the living worms incubated in 10 µM FBZ in the assay of living and killed worms. Furthermore, the formation of FBZSO_2_ showed a dose dependent formation.

We found that the motility of *T. suis* as compared to *O. dentatum* was less affected by increasing concentrations of FBZ and ALB. A low sensitivity to high concentrations of ALB has also been described for *T. muris* where doses up to 200 µg/ml (equivalent to 754 µM) of ALB were tested against adult and L3 stages of *T. muris in vitro* [9]. This dose level, which is approximately 25 times higher than the highest concentration used in our study (30 µM) did not reduce the motility of *T. muris* by 50% (IC_50_) after an incubation period of 72 hours. In contrast to *T. suis*, *O. dentatum* was found to be more sensitive to increasing concentrations of FBZ and ALB when incubated for 24 and 72 hours respectively. The high sensitivity towards increasing concentrations of ALB and FBZ has also been reported by Petersen et al. [41] who found that a concentration of 0.1 µM was able to inhibit migration of *O. dentatum* through a mesh by 61% for ALB and 69% for FBZ. An increase in concentration to only 3.16 µM increased the inhibition of migration to 75.3% for ALB and 76.2% for FBZ. The high sensitivity towards increasing concentrations of ALB and FBZ reported by Petersen et al. [41], is in agreement with our results *in vitro*, but more importantly, it is also in concordance with the high efficacy of FBZ against *O. dentatum* reported *in vivo* [20], [21]. Likewise, low sensitivity of *T. muris* towards ALB *in vitro* has also been shown to correlate with low treatment efficacy *in vivo* [9]. *Trichuris suis* was more sensitive towards increasing concentrations of LEV than *O. dentatum*. At the highest dose (200 µM) no movement of *T. suis* was observed neither after 24 or 72 hours incubation. A high sensitivity towards LEV has also been observed for *T. muris in vitro* (IC_50_ = 33.1 µg/ml equivalent to 68.5 µM) and *in vivo* where the worm burden was reduced by 95.9% with a single oral dose of LEV (200 mg/kg) in mice [9]. In pigs, the efficacy of a single oral dose of LEV (7.5–8 mg/kg) has shown varying efficacy on *T. suis* ranging from 26% [16] to 100% [48], [49].

In the *in vitro* assay with living and killed worms we found that the total concentrations of anthelmintic drugs were lower in *T. suis* than *O. dentatum* (Fig. 2). This applied to all three anthelmintics tested, although the difference was not found to be significant when living parasites were incubated in FBZ (Fig. 2). Incubation in increasing concentrations of FBZ and ALB, ranging from 0.01 to 30 µM for 72 hours revealed similar K_d_ values for *T. suis* and *O. dentatum* which suggests that approximately the same concentrations of FBZ and ALB are needed for both species in order to achieve binding of half of the binding sites at equilibrium. The B_max_ values were significantly lower for *T. suis* than *O. dentatum* suggesting that *T. suis* has a significantly lower binding capacity of FBZ and ALB than *O. dentatum* (Fig. 3, Table 1) which is in accordance with lower effect of these two anthelmintics on motility. The B_max_ values measured in *O. dentatum* were higher after 72 hours than 24 hours incubation. The accumulation of FBZ and ALB may be due to a lower secretion capacity of *O. dentatum*, in comparison to *T. suis*, which is supported by the formation of FBZSO_2_ in *T. suis*. The concentration of LEV within living worms were below the detection level of the HPLC analysis when incubated in 0.01, 0.1, and 1 µM, but interestingly the concentration of LEV within *T. suis* was more than two times higher than in *O. dentatum* when incubated in 200 µM LEV for 72 hours, which was translated into an absence of motor activity in the motility assay.

In the *in vitro* assay of living and killed worms we found that only living *T. suis* were able to metabolize FBZ, or possibly OXF, to the inactive metabolite FBZSO_2_ (Fig. 2), amounting 9.7% of the total anthelmintic concentration measured within the worms. When incubating the worms in increasing concentrations of FBZ for 24 hours we obtained inconsistent results for FBZSO_2_ (i.e. FBZSO_2_ was only detected in *T. suis*, and only when incubated in 1 µM FBZ) (data not shown). After 72 hours a concentration dependent formation of FBZSO_2_ was measured within *T. suis* where it represented between 6–17.2% of the total drug concentration whereas in *O. dentatum* it only constituted 0.8–0.9%. In relation to the maximum binding of FBZ, we measured a significantly lower value for *T. suis* than *O. dentatum* (Fig. 3 and Table 1). We therefore suggest that the poor effect of FBZ on *T. suis* may be related to a lower drug uptake and/or a higher detoxifying capacity of this species, however, some care should be taken with the latter. Albendazole and FBZ are able to undergo spontaneous oxidation to their corresponding derivatives ALBSO and OXF when mixed with DMSO [50]. The average proportions of the metabolites OXF and ALBSO were approximately the same within *T. suis* and *O. dentatum* when incubated in increasing concentrations of ALB and FBZ. Furthermore, these metabolites occurred in killed worms of both species and even trace amounts of ALBZSO_2_ were detected in killed *O. dentatum*. Therefore these findings indicate that OXF and ALBSO were formed by spontaneous oxidation, and that the formation of FBZSO_2_ observed in *T. suis* may be related to the presence and further transformation of OXF. As FBZSO_2_ were not detected in any of the killed worms or in living *O. dentatum* when incubated in 10 µM FBZ for 24 hours, it is most likely that the relative high concentrations of FBZSO_2_ measured in *T. suis* were not formed by spontaneous oxidation, but by *T. suis* itself. A trace amount of ALBSO_2_ (4.71 pmol/mg dry worm tissue) was measured in killed *O. dentatum* when incubated for 24 hours in 10 µM ALB but was not detected in any of the two species when incubated in increasing concentrations of ALB or in dead *T. suis*. Therefore it is most likely that occurrence of this compound is a detection uncertainty, which needs to be confirmed in future studies.

The above mentioned findings raise the following questions: a) why is the total drug concentrations of BZs generally lower in *T. suis* than *O. dentatum*? b) Why is the difference between concentration of anthelmintic within living and killed worms more pronounced for *O. dentatum* than *T. suis*? Considering the first question, possible entry routes of anthelmintic drugs into parasitic nematodes are oral ingestion or passive or active transport across the cuticle. In a study performed by Ho et al. [51], transport across the cuticle was demonstrated to be the main route of entry of lipophilic compounds (hydrocortisone and *p*-nitrophenol) into the nematode *A. suum* [51]. This route was confirmed by Mottier et al. [32] who also suggested that as a general rule helminths uptake BZs by passive diffusion [32]. Since previous work indicated that passive diffusion across the cuticle is the main route of uptake of lipophilic anthelmintics, and a transcuticular route also has been shown for the water soluble anthelmintic LEV [33], we therefore assumed that this also was the case for *T. suis* and *O. dentatum*. Oral ingestion of anthelmintic was controlled in the present study by killing the worms, but the concentration of all three anthelmintics was lower in *T. suis* than *O. dentatum* whether killed or alive, with the exception of living worms exposed to FBZ (Fig. 2). Furthermore, the binding capacity of *T. suis* was significantly lower than the binding capacity of *O. dentatum* when exposed to both FBZ (*P*<0.0001) and ALB (*P* = 0.033). The average proportions of the metabolites OXF and ALBSO were approximately the same for both species, whereas concentration levels above 5 pmol/mg dry tissue of FBZSO_2_ were only detected in *T. suis*. We therefore speculate that the lower total drug concentration of BZs measured both in living (i.e. B_max_ values after 72 hours incubation in ALB and FBZ) and killed *T. suis* may be due to structural differences in the cuticle or different lipid contents. Considering the second question regarding the different concentration of anthelmintic within living and killed worms, Mottier et al. [32] found that the concentration of FBZ was lower within living *A. suum* as compared to killed worms. These findings correspond to our observation for *O. dentatum* exposed to all three anthelmintics, although the difference was not significant when the worms were incubated in LEV (*P* = 0.09). For *T. suis*, a significantly lower concentration within living worms in relation to the killed, was only observed when exposed to ALB. The rate of drug diffusion across the cuticle of *A. suum* and other nematodes is restricted by the lipid barrier in the hypodermis, the p*K*_a_ of the drug, the pH of the aqueous environment within the cuticula and the negatively charged aqueous filled pores within the collagen matrix [52]. Mottier et al. [32] suggested that the lower concentration within living worms is related to the acidic environment at the nematode surface that is created by excretion of acidic organic metabolites from the worms [53]. Benzimidazoles are weak bases [54] and may therefore largely exist in their ionized form in the acidic environment at the nematode surface. The ionized form is not readily diffusible through the lipid layer of the cuticle therefore a smaller amount of BZs may enter the living parasites compared to the killed. This mechanism may be the reason why we observed a lower concentration of anthelmintic in living *O. dentatum*, and to a lesser extent in living *T. suis*, compared to the killed specimens.Nevertheless, damage of the cuticle due to freezing and a subsequent increase in permeability or possibly higher drug concentrations trapped in the cuticle of killed worms cannot be ruled out. Furthermore, inactivation of possible ATP-dependent efflux pumps i.e. the ATP-binding cassette (ABC) transporter P-glycoprotein (Pgp) [34], [55] may also contribute to the increased drug concentration observed within the killed worms. Interestingly, we did not observe the same for *T. suis* when exposed to FBZ and LEV which further supports our hypothesis that the lower drug concentration measured within this species is also related to a lower drug uptake.

An answer to the intriguing question for low to varied treatment efficacy of *T. trichiura* infections in humans has been sought from a variety of angles. The majority of these has taken an empiric approach by evaluating the effect of different treatment strategies in clinical trials such as: a) comparing the efficacy of single-dose BZs treatment (i.e. ALB (400 mg) and MBD (500 mg)) with the efficacy of combination therapy (i.e. BZs in combination with LEV (40 or 80 mg), ivermectin (200 µg/kg) or diethylcarbamazine (150 mg) [4], [5], b) comparing the efficacy of single-doses with triple-doses of ALB and MBD [6] or c) comparing the efficacy of single and double doses of ALB and MBD given alone or in combination [56]. In the above-mentioned clinical trials the highest CR (70.7%) was obtained using 3×500 mg MBD given over 3 consecutive days [6]. Empiric approaches have also been performed using *T. muris* as a model where the effect of single-drugs (i.e. monepantel, ALB, LEV, pyrantel pamoate and oxantel pamoate) and drug combinations between ALB, LEV, MBD, pyrantel pamoate, oxantel pamoate and ivermectin (IVM) have been assessed in both *in vitro* assays and *in vivo* studies [9], [57], [58]. Albendazole, given as a single-drug, showed poor effect *in vivo* (600 mg/kg) and low efficacy *in vitro* (50–200 µg/ml) [9], whereas the combinations of ALB-MBD, MBD-IVM, MBD-LEV and oxantel pamoate-MBD revealed a strong synergistic effect suggesting combination therapy as a future possibility [57]. Yet other approaches have been used in order to find explanations for low to mediocre treatment efficacy of BZs against *Trichuris* spp. infections. Specific variants of the beta-tubulin gene (i.e. single nucleotide polymorphisms (SNPs) in codon 167, 198 and 200) have been reported to convey BZ-resistance in parasitic nematodes of veterinary importance [59]–[63] and SNPs in codon 200 have been identified in *T. trichiura* obtained from a human population expected to be unexposed to BZs [64]. Furthermore, there is evidence demonstrating a higher frequency of the resistant genotype in codon 200 (TAC/TAC) in eggs of *T. trichiura* isolated from human populations in Haiti and Kenya after treatment with ALB [65], indicating that anthelmintic resistance may be involved in the low to mediocre treatment efficacy of BZs reported for this genus. However, such SNPs were not found in other *Trichuris* spp. [66], and not systematically in human populations [67].

The present work represents yet another approach to address the intriguing question for low to varied treatment efficacy of *T. trichiura* infections in humans. Based on worm motility, concentration of anthelmintic drugs and their metabolites within the worms and the difference in binding capacity of FBZ and ALB, we suggest that the lower sensitivity of *T. suis* towards these drugs *in vitro* is, in comparison to *O. dentatum*, due to a lower drug uptake. Furthermore, our data indicate that *T. suis* is able to transform FBZ or OXF into the inactive metabolite FBZSO_2_. Whether the drug uptake of *T. suis in vitro* mirrors the drug uptake *in vivo* is still unresolved. In the host, *Trichuris* spp. are attached to the mucosa with the anterior part which may give the worms a mechanical advantage in relation to anthelmintic treatment (they do not easily get detached even when temporarily deprived for energy or paralysed). Furthermore, such attachment mayserve as a protective barrier of the anterior part against active drugs in the intestinal lumen and instead render the worms more exposed to less potent anthelmintic metabolites in the blood. However, the posterior part is largely exposed to drugs in the lumen. We do not know whether the majority of the drug acting on *Trichuris* spp. comes from the intestinal lumen or whether it arrives via the blood supplying the intestine or both, but by using *T. suis* as a model we have shown that the varied and low drug efficacy against *Trichuris* spp. in animals and humans may be related to low drug-uptake in the worms.
